# Adherence and barriers to *H. pylori* treatment in Arctic Canada

**DOI:** 10.3402/ijch.v72i0.22791

**Published:** 2013-12-31

**Authors:** Megan Lefebvre, Hsiu-Ju Chang, Amy Morse, Sander Veldhuyzen van Zanten, Karen Jean Goodman, CANHelp Working Group

**Affiliations:** 1 Department of Public Health Sciences, University of Alberta, Edmonton, Alberta, Canada; 2 Department of Medicine, University of Alberta, Edmonton, Alberta, Canada

**Keywords:** Aboriginal health, *Helicobacter pylori*, peptic ulcers, cancer, circumpolar regions

## Abstract

**Introduction:**

*Helicobacter pylori* infection is an emerging health concern to some northern Canadian Aboriginal communities and their clinicians. Clinicians in the north perceive *H. pylori* infection to be a major clinical problem because they find *H. pylori* infection in many patients evaluated for common stomach complaints, leading to frequent demand for treatment, which often fails. Moreover, public health authorities identified the need for information to develop locally appropriate *H. pylori* control strategies. We described adherence and identified barriers to completing treatment among *H. pylori*-positive participants in a community-based project inspired by local concerns about *H. pylori* infection risks.

**Methods:**

In 2008, 110 *H. pylori*-positive participants (diagnosed by a breath test, histopathology and/or culture) of the Aklavik *H. pylori* project were randomised to standard-of-care or sequential treatment. We ascertained adherence by interviewing participants using a structured questionnaire. We estimated adherence frequencies as the proportion of participants who reported taking either 100% of doses (perfect adherence) or ≥80% of doses (good adherence). To compare the proportion with perfect or good adherence in subgroups, we report proportion differences and 95% confidence intervals (CI).

**Results:**

Of 87 participants who were interviewed, 64% reported perfect adherence and 80% reported good adherence. We observed more frequent perfect adherence for: standard therapy (67%) versus sequential (62%); males (76%) versus females (52%); participants 40–77 years (79%) versus 17–39 (50%). Proportion differences were 5% (CI: −15, 25) for standard versus sequential therapy; 23% (CI: 4, 43) for male versus female; and 29% (CI: 10, 48) for 40–77 versus 15–39 years for perfect adherence. Of the 29 participants who reported poor adherence (<80% of doses taken), the following barriers to treatment were reported: changed mind about taking treatment (24%), consumption of alcoholic beverages (18%), nausea (18%), forgetfulness (12%), stomach pain (12%), difficulty in swallowing pills (6%), no reason (6%) or bad taste of the pills (6%).

**Conclusion:**

This analysis suggests that adherence to treatment for eliminating *H. pyori* infection may vary by regimen and may be influenced by socio-demographic factors. These findings add to the small body of evidence pertaining to adherence to *H. pylori* treatment in Arctic Aboriginal communities. On-going research in additional northern Canadian communities will accumulate data for developing recommendations to improve adherence for treatment to eliminate *H. pylori* infection.

Chronic *Helicobacter pylori* infection increases the risk of peptic ulcer disease and gastric cancer, and has been estimated to affect half or more of the world's population (1) The low prevalence of *H. pylori* infection in Canadian children suggests that current levels of *H. pylori* transmission are low in major Canadian urban centres (2). There are still a sizable number of Canadians, however, who harbour active *H. pylori* infections acquired in earlier eras or other parts of the globe. Furthermore, some settings in northern Canada have suboptimal living conditions where endemic *H. pylori* transmission appears to continue.

Despite limited data on the occurrence of *H. pylori* infection in northern Canada, this infection has become a health concern for members of northern communities and their healthcare providers. Estimates of *H. pylori* sero-prevalence range from 50 to 95% in Aboriginal communities in northern Canada, 2–3 times higher than the Canadian average (3), and the incidence of stomach cancer is increased in the Northwest Territories (NWT) compared to the rest of Canada (3). Moreover, in NWT communities, where *H. pylori* infection is frequently diagnosed, there is growing awareness that this infection is a risk factor for stomach cancer (3). *H. pylori* infection is a challenging clinical problem for primary healthcare providers in northern communities because they find the infection in many patients evaluated for common stomach complaints, thus leading to frequent need for treatment, which often fails for unknown reasons.

Public health authorities have identified the need for information to develop locally appropriate *H. pylori* control strategies for optimal allocation of healthcare resources aimed at reducing the *H. pylori*-induced disease burden. At the request of concerned communities and their healthcare providers, the Canadian North *Helicobacter pylori* (CAN*Help*) Working Group was formed to link University of Alberta researchers with northern health authorities and community organisations. This group works together towards the following common goals: (a) to obtain representative data from diverse settings in northern Canada for informing regional public health strategies for reducing *H. pylori* infection; (b) to develop knowledge-exchange strategies that help community members understand *H. pylori* health risks as well as currently available solutions and unsolved challenges for reducing these health risks; and (c) to conduct policy analysis to identify cost-effective *H. pylori* management strategies that are ethically, culturally and economically appropriate for northern communities.

A major challenge for potential public health strategies aimed at reducing health risks from *H. pylori* infection stems from the difficulty in identifying antibiotic therapies that are effective at eliminating this infection across populations. Complex multidrug therapies are required to eliminate this infection. The effectiveness of the most successful of these therapies is known to vary widely across geographic locations and, in general, to be lowest in regions where prevalence is highest (4). Clinical trial results show that adherence to the prescribed regimen is a key determinant of *H. pylori* treatment success (5–7). There is little evidence on the effectiveness of treatment to eliminate *H. pylori* infection in northern Aboriginal peoples or on adherence to such therapies in the circumpolar north. One study conducted in Alaska reported that among 96 patients who had a *H. pylori* breath test 8 weeks after treatment, 29% tested *H. pylori*-positive (8). Initial results from treatment trials conducted by the CAN*Help* Working Group show that commonly used triple drug and sequential regimens fail to eliminate *H. pylori* infection in 30–40% of trial participants (unpublished data). To better understand why *H. pylori* regimens appear to have limited success in northern regions, this analysis uses data from research carried out by the CAN*Help* Working Group to address the following specific aims: (a) to estimate adherence frequencies in a northern Canadian population for standard-of-care and alternative *H. pylori* therapies; (b) to assess treatment effectiveness in relation to adherence frequencies; and (c) to identify barriers to completing *H. pylori* treatment in the context of community-based participatory research.

## Methods and materials

### Study setting

This study uses data from the treatment trial component of the Aklavik *H. pylori* Project (9), the initial project carried out by the CAN*Help* Working Group, in the Hamlet of Aklavik, NWT (population ~600), where 90% of the population is either Inuvialuit (Inuit) or Gwich'in Dene (First Nations). The Aklavik *H. pylori* Project aims to collect data on the burden of disease and risk factors associated with *H. pylori* infection, and develop knowledge-exchange strategies to address community concerns. Fieldwork for this on-going project began in November 2007 and all residents of Aklavik were invited to participate. As of June 2012, 384 residents had participated as follows: 345 completed health surveys; 333 had C13-urea breath tests (UBT); and risk factor surveys were collected from 285 individuals and 145 households. Among participants screened by UBT, 58% (194/333) were positive for *H. pylori* infection. In February 2008, all Aklavik residents regardless of *H. pylori* status were offered upper gastrointestinal endoscopy and biopsies were obtained from 194 for bacterial culture and antibiotic susceptibility testing. (These were not all the same 194 participants who were UBT positive.)

### Treatment trial

#### Design

We conducted a single-centre, randomised controlled trial during November 2008–April 2009. Ethical approval was granted by the NWT research licensing institute and the University of Alberta Health Research Ethics Board. Blinding the investigators or participants to the allocated treatment was not feasible due to difficulties in masking complexities of the regimens, which used differing numbers of drugs and varied on whether the drugs were taken concurrently or sequentially. As part of the Aklavik *H. pylori* Project, project gastroenterologists assessed eligibility and treatments were prepared in bubble packs by a commercial pharmacy in the region. Project staff supported by the nursing staff of the Aklavik Health Centre disbursed assigned treatments. Participants were asked to return the bubble packs to the local health centre with any skipped doses upon treatment completion. At that time, local nursing staff recorded the number of doses completed according to the returned bubble packs and administered a post-treatment questionnaire to ask about treatment adherence and difficulties encountered in completing the treatment. We ascertained *H. pylori* status by UBT at least 10 weeks post-treatment. Project staffs who analysed post-treatment breath tests were not aware of which treatment each participant received.

#### Eligibility

All Aklavik *H. pylori* Project participants ≥15 years old who tested positive for *H. pylori* infection by UBT, histopathology or culture between 2008 and 2009 were candidates for the treatment trial. Participants received information regarding the pros and cons of treatment for *H. pylori*-positive persons without symptomatic disease to ensure informed decision making. Exclusion criteria included: (a) allergies to study medications; (b) severe cardiorespiratory, pulmonary, endocrine, hepatic or renal disease; and (c) pregnant or lactating. We offered appropriate therapy and follow-up testing to *H. pylori-*positive participants who did not wish to participate in the trial or who did not meet eligibility criteria (including children <15 years of age).

#### Treatments

**Fig. 1:**
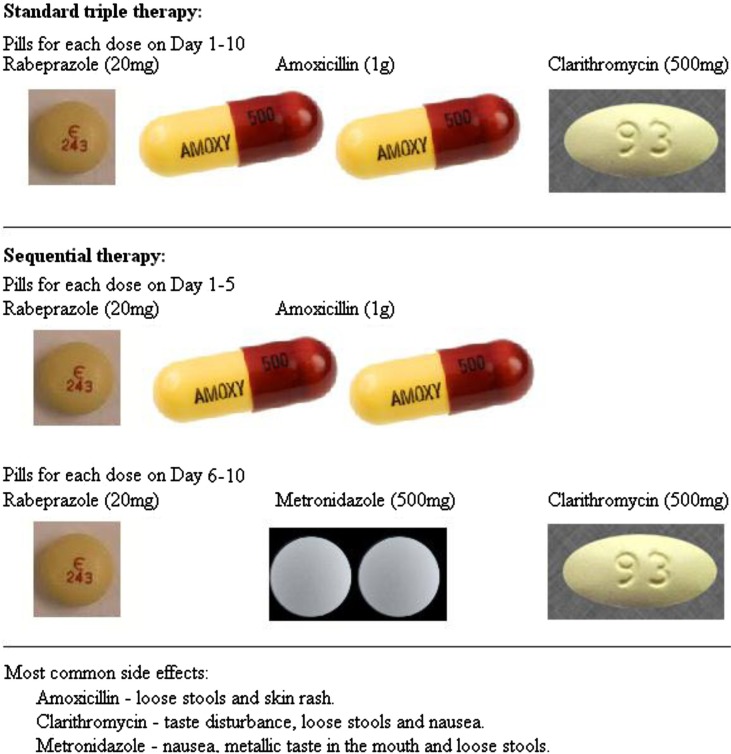
Pills consumed for each dose of standard triple therapy and sequential therapy.

Standard-of-care *H. pylori* treatment regimens used worldwide combine 2–3 antibiotics with a proton-pump inhibitor (10). The current standard first-line treatment in Canada includes amoxicillin, clarithromycin and lansoprazole for 7–10 days in a single, combined-drug daily sustained-release dose. This trial used the following treatment regimens, each of which was administered for 10 days: (a) standard triple therapy: twice-daily oral doses of rabeprazole (20 mg), amoxicillin (1 g) and clarithromycin (500 mg); or (b) sequential therapy: twice-daily oral doses of rabeprazole (20 mg) and amoxicillin (1 g) for days 1–5 followed by twice-daily oral doses of clarithromycin (500 mg) and metronidazole (500 mg) for days 6–10. Figure 1 shows the pills consumed for each dose of each of the 2 regimens. (An alternate quadruple regimen was assigned to 3 of the trial participants, but none of the 3 were included in this analysis due to missing adherence data, thus details on this regimen are not presented.) Participants who were assigned therapy including metronidazole were instructed to avoid alcohol while on this drug. Regardless of the assigned regimen, all participants were told it was preferable to stop consuming alcohol while on the treatment to facilitate remembering to take pills as prescribed.

#### Randomisation

Participants were assigned a random number upon entering the trial and the treatment allocation depended on whether the assigned number was odd or even. Treatment naïve participants with *H. pylori* strains that tested susceptible to clarithromycin or that were not tested for antibiotic susceptibility were randomly allocated either the standard-of-care regimen or the sequential regimen. Participants who previously failed treatment or whose *H. pylori* strains tested not susceptible to clarithromycin were randomly allocated either the sequential regimen or the quadruple regimen mentioned above.

#### Adherence

Participants received treatments in bubble packs so that pills were grouped according to the days and times the pills were to be taken to facilitate adherence to the regimen. We gave participant's information that emphasised the importance of: adherence to the treatment regimen; leaving skipped doses in the bubble pack; returning the bubble pack when they finished the treatment; and having a post-treatment test to see if their infection cleared. Our project staff attempted to phone or visit participants daily during the treatment period, to remind participants about taking pills at the designated times, monitor adherence and actively ascertain adverse effects or obstacles to adherence. Project staff undertook rigorous follow-up measures for maximising participation in post-treatment breath tests and collection of used pill packs to minimise missing data on outcomes and adherence. In June 2010 and November 2011, project staff attempted to contact participants with missing adherence data to conduct post-treatment interviews.

### Outcome measures

Adherence to *H. pylori* treatment was the main outcome of this analysis. Classification of treatment adherence was primarily based on self-reported data and supplemented by the pill counts from returned bubble packs. Data obtained from pill counts can be a misrepresentation of how many pills participants actually took, since pills can be thrown out or given to other people. Also, participants who failed to return bubble packs to the local health centre could be interviewed by telephone to obtain adherence information. This revealed that many of the participants who did not return their bubble packs had not completed their treatment. For these reasons, self-reported data were favoured. Thus, as the primary data source, participants were asked to estimate how many pills they took. In addition, participants were asked the number of days they adhered to the treatment and a percentage was calculated from this. If no self-reported data were available, a percentage was calculated from the pill counts from returned bubble packs. Adherence was classified as perfect (100%), good (≥80%) or poor (<80%). The decision to define good adherence as ≥80% was based on the standard cut-off value utilised in adherence research (11–13). Our interviewers asked participants who reported missing doses to explain why they did not complete all of their prescribed medication, taking care to avoid being judgemental about non-adherence by acknowledging that people often have difficulty in taking pills for various reasons and explaining that the research project aimed to find out what these difficulties were so that solutions could be identified.

### Statistical analysis

We describe participant characteristics by treatment group using mean and standard deviations for continuous variables and proportions for categorical variables. We estimated adherence frequencies as the proportion of participants who reported taking 100% (perfect adherence) or ≥80% (good adherence) of prescribed doses. To compare the proportion of participants with perfect and good adherence across categories of treatment regimen, age, sex and education, we estimated proportion differences and 95% confidence intervals (CI) using the “prtest” command in the STATA 12 software package. We estimated frequencies of barriers to treatment as the proportion of participants with poor adherence who mentioned a particular barrier.

## Results

Of 110 trial participants prescribed either the standard or sequential treatment, adherence data were available for 87 (79%). Table I shows similar socio-demographic distributions in the 110 trial participants and the 87 with complete data. Among the 87 participants included in this analysis, there were similar proportions of males and females, with an average age of 40 years; most identified as Inuvialuit (western Canadian Inuit) and reported having an education of ≤grade 12. As expected, similar proportions of participants were prescribed standard or sequential therapy.

**Table I:** Demographics of participants in the trial component of the Aklavik *H. pylori* Project, 2008–2011

	Trial participants n=110		Trial participants with adherence data n=87	
		
Characteristic	Number	Percent	Number	Percent
Treatment regimen				
Standard	53	48	45	52
Sequential	57	52	42	48
Sex				
Male	54	49	45	52
Female	56	51	42	48
Age (years)				
15–39	53	48	44	51
40–73	57	52	43	49
Ethnicity				
Gwich'in First Nations	32	29	27	31
Inuvialuit (Inuit)	63	57	50	57
Non-Aboriginal	8	7	4	5
Other Aboriginal	7	6	6	7
Education				
≤Grade 12	88	80	70	80
>Grade 12	22	20	17	20

### Adherence

Perfect adherence was reported by 64% of participants, while another 16% were in the good adherence category (≥80%) and 20% reported poor adherence (<80%). Table II shows that a higher proportion of males, older participants and those with higher education levels reported perfect adherence, although all of these differences are notably smaller in the good adherence category. No clear difference in adherence was noted for the 2 treatment regimens.

**Table II:** Adherence frequencies by selected factors in 87 Aklavik *H. pylori* Project treatment trial participants, 2008–2011

	Perfect adherence (100% of doses taken)		Good adherence (≥80% of doses taken)	
		
	Adherence frequency (%)	Proportion difference (95% CI)	Adherence frequency (%)	Proportion difference (95% CI)
Sex				
Male	76	23%	87	5%
Female	52	(4%, 43%)	82	(−9%, 18%)
Age (years)				
5–39	50	29%	77	14%
40–77	79	(10%, 48%)	91	(4%, 27%)
Treatment regimen				
Standard	67	5%	81	7%
Sequential	62	(−15%, 25%)	88	(−20%, 7%)
Education				
≤Grade 12	60	22%	83	8%
>Grade 12	82	(1%, 44%)	91	(−6%, 22%)

### Barriers to adherence

Of the 17 participants who reported poor adherence, the proportion reporting the following barriers to treatment were: changed their mind about taking treatment (n=4), consumption of alcoholic beverages (n=3), nausea (n=3), forgetfulness (n=2), stomach pain (n=2), difficulty in swallowing pills (n=1), bad taste of pills (n=1) and no reason (n=1) (see Table III).

**Table III:** Reported barriers for not achieving 80% adherence among 17 Aklavik *H. pylori* Project treatment trial participants with poor adherence

Reason	Number	Percent of participants reporting barriers
Changed mind about taking treatment	4	24
Consumed alcoholic beverages	3	18
Nausea	3	18
Stomach pain	2	12
Forgetfulness	2	12
Difficulty in swallowing pills	1	6
Bad taste of pills	1	6
No reason	1	6

### Adherence and treatment success

Among participants with adherence data, 63% of 79 with known post-treatment *H. pylori* status were negative after treatment. Treatment effectiveness by adherence status was 70% (95% CI: 58–81%) among participants with good adherence (≥80%) and 33% (95% CI: 12–62%) among participants with poor adherence (<80%).

### Bias analysis

To assess the sensitivity of the results to the adherence data source, adherence estimates based on self-report were compared to adherence estimates based on pill counts from returned bubble packs. Using pill count data only, adherence estimates were all over 80%; no pill count data were obtained from participants who reported poor adherence (Table IV).

**Table IV:** Comparison of adherence estimated by pill counts and self-report among 110 Aklavik *H. pylori* Project treatment trial participants

	Adherence estimated by pill count from returned bubble packs			
		
Adherence estimated by self-report on post-treatment interview	Did not return bubble pack	Poor adherence (<80%)	Good adherence (≥80%)	Total
Did not complete questionnaire	24	0	3	27
Poor adherence (<80%)	13	0	4	17
Good adherence (≥80%)	28	0	38	66
Total	65	0	45	110

## Discussion

This analysis was part of a larger research project carried out in Aklavik, NWT, located in an Arctic region of Canada, where the prevalence of *H. pylori* infection is elevated (3). Our trial was conducted at the request of concerned Aklavik community members and their healthcare providers to identify optimal *H. pylori* therapies, treatment adherence frequencies and barriers to optimal adherence. This analysis examined 2 definitions of adherence: (a) perfect, defined as taking 100% of prescribed doses; and (b) good, according to the conventional classification of ≥80%, which implies that <80% of doses taken is poor adherence. According to the conventional definition, 80% of 87 Aklavik treatment trial participants with data reported good adherence. We observed that good adherence was substantially more frequent in older participants. We observed more striking associations between selected socio-demographic factors and perfect adherence; perfect adherence was more frequent in men and participants with higher education levels, as well as in older participants. We found participants stated the following reasons for poor adherence: changed their mind about taking the treatment, consumption of alcoholic beverages, nausea, forgetfulness, stomach pain, difficulty in swallowing pills and bad taste of pills. It should be noted that each of these specific obstacles was identified as such by fewer than 5% of trial participants with information on adherence.

Most medical therapies require a high level of adherence to obtain successful treatment outcomes. Studies have consistently shown that poor adherence is a major determinant of treatment success, and that physicians often fail to recognise poor adherence. As a result, understanding determinants of poor adherence is a major research focus. Our analysis shows that treatment success was strongly associated with adherence. Our results are in accordance with a systematic review of 561 trials published during 1984–1999, which found that *H. pylori* treatment success substantially depended on adherence to the regimen (14). Similarly, based on a meta-analysis of 98 trials published during 1990–2004, Fischbach et al. reported that adherence to quadruple *H. pylori* regimens ranged from 85 to 100% (15), and achieving good adherence contributed to treatment success. In a recent randomised treatment trial of 219 *H. pylori*-infected, iron-deficient, rural Alaskan children treated with iron sulfate alone or in combination with a 2-week course of lanzoprazole, clarithromycin and amoxicillin, treatment success was associated with perfect adherence (16).

The World Health Organization defines adherence as “the extent to which a person's behaviour – taking medication, following a diet, and/or executing lifestyle changes, corresponds with agreed recommendations from a healthcare provider” (7, p. 3). Recognised determinants of adherence correspond to 5 categories: treatment characteristics (e.g. regimen complexity, pill burden, side effects), condition characteristics (e.g. rate of progression of disease, disease severity), patient characteristics (e.g. substance or alcohol abuse, depression, age), healthcare team and system-related factors (e.g. the patient–provider relationship and characteristics of the medical system) and social and economic factors (e.g. social support, attitude and beliefs towards treatment, and income) (6). In our study, among participants found to have poor adherence, treatment-related (e.g. nausea, stomach pain) and patient-related factors (e.g. not wanting to take treatment, alcohol consumption) were most commonly reported as barriers to adherence. Treatment-related factors are the most commonly cited barriers to adherence across many medical therapies (7), thus it is not surprising that nausea and stomach pain were reported as a barrier to achieving perfect adherence. Problems arising from alcohol consumption are a challenging health issue in Canadian Aboriginal communities (17) and it may be that in addition to being acknowledged as a reason for poor adherence among trial participants, it discouraged other Aklavik residents from participating in the trial. It should also be noted that adherence is simultaneously influenced by multiple factors, and the ability to optimally follow a treatment plan is often compromised by more than 1 barrier. Solving the problems related to all dimensions of adherence is necessary if adherence to *H. pylori* therapy is to be improved.

This analysis may be limited by the imperfect ability of participants to accurately recall the number of missed doses of medication, a potential limitation in any study that relies on the memory of informants. As a result, we compared adherence estimates based on self-report to adherence estimates based on pill counts from returned bubble packs. We found that adherence estimates using pill count data were all over 80%; of the 17 participants who reported poor adherence, 13 did not return their bubble packs and the remaining 4 returned bubble packs with fewer than 20% of doses remaining. Given that falsely reporting poor adherence is unlikely, it seems that participants were reluctant to return bubble packs containing many unconsumed doses, perhaps due to fear of being judged as noncompliant. These observations suggest that ascertaining adherence from returned pill packages is likely to overestimate adherence and that non-judgemental interviews about difficulties encountered in taking medications may obtain more accurate estimates of adherence. Although we predominately measured adherence using self-report, we supplemented this information with pill count data to reduce missing data. Recently, Liu et al. (18) demonstrated that 3 adherence measures (electronic drug monitoring, pill counts and self-report) all have limitations that may be reduced by utilising a combined measurement approach.

Despite the limitations of this analysis, in particular, the uncertain accuracy of adherence classifications and insufficient data for some subgroup analyses of interest, our findings make an important contribution to the literature given the scarcity of information available for Canada's northern communities. Although researchers and healthcare workers acknowledge the seriousness and urgency of problems associated with treatment adherence, and adherence issues are increasingly discussed in leading health journals, little adherence research on *H. pylori*-infected residents of northern Canada has been published. Most *H. pylori* treatment research focuses on treatment effectiveness and microbial resistance. This analysis focused specifically on adherence to *H. pylori* medication and reasons for not achieving good adherence in a setting where *H. pylori* prevalence is high using a research design with a high level of community participation. As such, it improves current knowledge of issues surrounding adherence to *H. pylori* treatment in an Arctic Aboriginal setting.

Our community-based research arose from a unique situation in which affected communities identified *H. pylori* infection as a health research priority and sought input from scientists to address concerns about *H. pylori*-associated health risks. A major obstacle to reducing these health risks is the limited effectiveness of *H. pylori* treatment, widely shown to be least effective among those most at risk (4, 19). Among residents of communities that participate in CAN*Help* Working Group *H. pylori* projects, current clinical guidelines, derived from populations that respond better to treatment, result in an unacceptably high level of treatment failure. This analysis, along with forthcoming findings on treatment effectiveness and determinants of treatment success from our on-going projects in additional communities, aims to support recommendations by Fischbach et al. (4) to help healthcare providers in northern Canada learn more about “what works locally.”

In conclusion, future research on the effectiveness of treatment to eliminate *H. pylori* in populations at high risk of health consequences from this infection should aim to accumulate evidence on determinants of good adherence. Healthcare providers in such populations should carefully consider effective ways to facilitate completion of the complex treatment regimens, which patients may find daunting due to a variety of challenges. To this end, it may be beneficial to prepare those being prescribed treatment with strategies for coping with such things as the number of pills, as well as potential side effects and interactions with other substances. Our on-going research will add data from additional communities in the region to help identify potential interventions for facilitating adherence on treatments aimed at eliminating *H. pyori* infection.
